# Beyond motor neurons: autonomic dysfunction and ECG findings in adults with 5q-spinal muscular atrophy

**DOI:** 10.1007/s00415-025-13446-w

**Published:** 2025-10-14

**Authors:** Kaan Bacara, Stevan D. Stojanovic, Camilla Wohnrade, Alma Osmanovic, Olivia Schreiber-Katz, Aiden Haghikia, Susanne Petri, Bogdan Bjelica

**Affiliations:** 1 Department of Neurology, Hannover Medical School, 1, Carl-Neuberg-Strasse, 30625 Hannover, Germany; 2 Department of Cardiology and Angiology, Hannover Medical School, Carl-Neuberg-Strasse 1, 30625 Hannover, Germany; 3 PRACTIS Clinician Scientist Program, Dean’s Office for Academic Career Development, Hannover Medical School, Hannover, Germany; 4 Essen Center for Rare Diseases (EZSE), University Hospital Essen, Essen, Germany

**Keywords:** Spinal muscular atrophy, Multisystem disorder, Autonomic dysfunction, ECG, Quality of life

## Abstract

**Background:**

5q-Spinal muscular atrophy (SMA) is a lower motor neuron disease. However, peripheral organ involvement might contribute to its complex clinical picture, especially in severe forms. This study evaluated frequency and characteristics of autonomic symptoms, prevalence of electrocardiographic (ECG) abnormalities, and the impact of autonomic dysfunction on quality of life (QoL) in adults with SMA.

**Methods:**

Autonomic function was assessed using the SCales for Outcomes in PArkinson’s disease-Autonomic Dysfunction (SCOPA-AUT). 12-Lead ECG was recorded. The Hammersmith Functional Motor Scale Expanded (HFMSE), Revised Upper Limb Module (RULM), Fatigue Severity Scale (FSS), Beck Depression Inventory (BDI), and 36-Item Short Form Health Survey (SF-36) were collected to assess motor function, fatigue, depression, and QoL.

**Results:**

Forty-three SMA patients (23 males; mean age: 37.6 ± 14.1 years) and forty-three age- and sex-matched healthy controls were included. Compared to healthy controls, SMA patients scored worse on gastrointestinal domain (2.9 ± 2.9 vs. 1.1 ± 1.5, *p* < 0.001) and in total SCOPA-AUT score (9.8 ± 7.3 vs. 5.7 ± 4.4, *p* = 0.002). More severely affected patients reported a greater burden of autonomic symptoms compared to those with milder phenotypes. Burden of autonomic symptoms correlated with QoL (*ρ* = − 0.33, *p* = 0.028). The presence of major ECG abnormalities showed a negative correlation with SMA type (*ρ* = − 0.39, *p* = 0.027), age at the time of testing (*ρ* = − 0.45, *p* = 0.009), and ambulatory status (*ρ* = − 0.39, *p* = 0.027).

**Conclusion:**

Adults with SMA demonstrate a substantial burden of autonomic symptoms, especially those with more severe disease. This burden significantly compromises patients’ QoL. Further studies are needed to better understand autonomic dysfunction in this population.

**Supplementary Information:**

The online version contains supplementary material available at 10.1007/s00415-025-13446-w.

## Introduction

5q-Spinal muscular atrophy (SMA) is an autosomal recessive neuromuscular disorder characterized by degeneration of alpha motor neurons in the spinal cord and brain stem [1]. In about 95% of cases, SMA is caused by homozygous deletion of the *survival of motor neuron (SMN) 1* gene on chromosome 5q13.2 [2]. A paralogous *SMN2* gene can produce only about 10% of functional SMN protein due to alternative splicing of its pre-mRNA transcript, resulting in exclusion of exon 7. This is, however, insufficient to maintain normal motor neuron function. SMN protein is ubiquitously expressed in all tissues [3]. In complex with several other proteins, SMN forms a multi-protein complex that is involved in the assembly of mRNA splicing machinery [4]. Lee et al. emphasized the extra-neuronal role of the SMN protein and argued that systemic restoration is required to achieve effective treatment in severe SMA [5]. Hua et al. investigated the differential effects of systemic vs. central nervous system-specific restoration of SMN in a severe SMA mouse model using antisense oligonucleotides administered either intracerebroventricularly or subcutaneously. Systemic delivery in neonatal mice resulted in a significantly more robust phenotypic rescue, underscoring the critical role of SMN in peripheral tissues in severe SMA [6].

Although SMA has been primarily considered a motor neuron disease, these findings along with other growing preclinical and clinical evidence suggest that peripheral organ involvement significantly contributes to its complex clinical picture [7]. The autonomic nervous system may also be compromised as a consequence of SMN deficiency. However, clinical studies investigating autonomic dysfunction in SMA remain limited and have predominantly focused on infants and children [8–10]. To date, the burden of autonomic symptoms in adults with SMA has not been systematically investigated. With the emergence of new therapies that significantly prolong survival [11], addressing this gap has become increasingly relevant.

The aims of this study were to assess the prevalence and severity of autonomic symptoms and ECG (electrocardiogram) abnormalities in adults with SMA compared to healthy controls (HC), and to evaluate the impact of autonomic dysfunction on quality of life (QoL).

## Methods

This cross-sectional study included 43 SMA patients, treated at the Department of Neurology, Hannover Medical School, between April 2018 and July 2024. The study cohort comprised SMA patients aged 18 years or older who either had homozygous deletion of exons 7 and/or 8 of the *SMN1* gene or a compound heterozygous deletion involving exons 7 and/or 8 of one *SMN1* allele, along with a different pathogenic variant on the second allele. Additionally, 43 age- and sex-matched HC, recruited from the authors’ direct environment, were enrolled in the study. The following sociodemographic and clinical data were obtained: sex, age at the time of testing, body mass index (BMI), disease duration, SMA type, ongoing disease-modifying therapy, *SMN2* copy number, walking ability (defined as the ability to walk at least 10 m without assistance or use of a device such as cane or a walker [12]), presence of scoliosis, spondylodesis, use of non-invasive ventilation (NIV) and percutaneous endoscopic gastrostomy (PEG). BMI was calculated as body weight (in kilograms) divided by height squared (in meters). The study was approved by the Ethical Board of Hanover Medical School (no. 6269) and all individuals gave their written informed consent to participate.

### Motor function assessment

Motor function was assessed by trained physiotherapists using two scales: the Revised Upper Limb Module (RULM) and the Hammersmith Functional Motor Scale Expanded (HFMSE). The RULM is a disease-specific score consisting of 20 items that tests the motor ability of the upper extremities [13]. The maximum possible score on RULM is 37, where higher scores denote better function. The HFMSE is a disease-specific score that tests gross motor function. It comprises 33 items, with a maximum total of 66 points, where highest scores reflect better motor function [14].

### Assessment of the autonomic function

To assess the autonomic function, the German version of the SCales for Outcomes in Parkinson’s disease-Autonomic Dysfunction (SCOPA-AUT) questionnaire was utilized. This instrument evaluates autonomic symptoms through 23 items, each scored on a scale from zero to three, with zero indicating “never experiencing a symptom” and three indicating “often experiencing a symptom”. The questionnaire comprises six domains and yields a maximum score of 69, where higher scores denote more severe autonomic dysfunction. Domains include gastrointestinal, urinary, cardiovascular, thermoregulatory, pupillomotor and sexual [15]. The authors obtained permission for the use of German version of the SCOPA-AUT, as approved by the Movement Disorder Society.

### Electrocardiogram

Patients underwent standardized 12-lead ECG derived from ten surface electrodes: four limb electrodes (placed on the right and left arm and legs) and six precordial electrodes (V1–V6) placed in the anterior thorax. ECG recordings were obtained with the patient in a supine or sitting position, using a standard paper speed of 25 mm/s and a calibration of 10 mm/mV. The abnormal findings were categorized as “major” and “minor”, according to the study of Yagi et al. [16]. Supplemental Table 1 provides a modified version of the classification system of ECG abnormalities, adapted from Yagi et al. [16]. Eight patients declined to participate in this part of the study, and three ECG recordings were of insufficient quality, leaving thirty-two ECGs available for further analysis.

### Assessment of depression, fatigue, and quality of life

To assess depression, the Beck Depression Inventory (BDI) was used, which consists of 21 items. Each item is scored from zero to three. A total score of 11 or higher is considered indicative of depression [17].

To evaluate fatigue, the Fatigue Severity Scale (FSS) was administered. This score comprises nine items, where each item is rated from one to seven, yielding a maximum total score of 63 (18). Significant fatigue was defined as a score of 36 or higher [18].

To assess QoL, the 36-Item Short Form Health Survey (SF-36) was used. This questionnaire comprises 36 items grouped into 8 domains: physical functioning (PF), role physical (RP), bodily pain (BP), general health (GH), vitality (VT), social functioning (SF), role emotional (RE), and mental health (MH). Each domain is scored from 0 to 100, with higher scores indicating better QoL. The domain scores are further summarized into two composite scores, the physical component score (PCS) and the mental component score (MCS), as well as an overall SF-36 total score [19].

### Statistical analysis

Statistical analysis was conducted using IBM® SPSS® (version 28, Chicago IL, USA). Data normality was assessed with the Shapiro–Wilk and Kolmogorov–Smirnov tests, along with graphical inspection of histograms. Continuous variables were reported as mean and standard deviation (SD) or median and interquartal range. Group comparisons were performed using the ${x}^{2}$ test, Fisher’s exact test, Mann–Whitney U test, or Student’s test, as appropriate. Correlations were determined with Spearman’s rank (correlation) coefficient or Phi coefficient, as appropriate. A *p* value < 0.05 was considered statistically significant, while *p* < 0.01 indicated high statistical significance.

## Results

### Participants’ characteristics

The baseline sociodemographic and clinical characteristics of the enrolled SMA patients and HC are summarized in Table 1. There were no significant differences between the groups regarding BMI. The majority of SMA patients had SMA type 2 or 3, were non-ambulatory, and were receiving disease-modifying therapy at the time of assessment. Only three SMA patients reported current smoking.

**Table 1:** Main sociodemographic and clinical characteristics of enrolled SMA patients and HC

Features	SMA patients	HC
*N*	43	43
Male gender (*N*, %)	23, 53.5	22, 51.2
Age at testing		
Years, mean ± SD	37.6 ± 14.1	37.3 ± 14.1
Years, median [min–max]	36.5 [18.0–68.0]	36.5 [18.0–67.0]
BMI		
kg/m^2^, mean ± SD	23.1 ± 7.6	25.4 ± 4.7
kg/m^2^, median [min–max]	22.6 [9.0–36.7]	24.5 [17.8–40.3]
Disease duration		–
Years, mean ± SD	31.4 ± 13.9	
Years, median [min–max]	32.0 [5.0–66.5]	
SMA type (*N*, %)		–
Type 1	1, 2.3	
Type 2	20, 46.5	
Type 3	21, 48.9	
Type 4	1, 2.3	
Ongoing therapy (*N*, %)		–
Nusinersen	26, 60.5	
Risdiplam	14, 32.5	
No therapy	3, 7.0	
*SMN2* copy number		–
Mean ± SD	3.5 ± 0.8	
Median [min–max]	3.0 [2.0–6.0]	
*SMN2* copy number		–
< 4	22, 51.2	
≥ 4	17, 39.5	
Missing	4, 9.3	
Walking ability (*N*, %)		–
Ambulatory	12, 27.9	
Non-ambulatory	31, 72.1	
Scoliosis (*N*, %)	32, 76.2	–
Spondylodesis (*N*, % out of all patients with scoliosis)	12, 37.5	–
NIV (*N*, %)	9, 20.9	–
PEG (*N*, %)	2, 4.9	–
HFMSE score		–
Mean ± SD	18.5 ± 21.9	
Median [min–max]	5.5 [0.0–63.0]	
RULM score		–
Mean ± SD	19.5 ± 12.1	
Median [min–max]	17.0 [0.0–37.0]	
BDI score		–
Mean ± SD	7.5 ± 8.3	
Median [min–max]	5.0 [0.0–39.0]	
% of depressed	10, 23.2	
FSS score		-
Mean ± SD	42.6 ± 12.1	
Median [min–max]	44.0 [12.0–61.0]	
% of fatigued	29, 67.4	
Total SF-36 score		–
Mean ± SD	60.4 ± 16.2	
Median [min–max]	61.3 [15.9–91.6]	

SMA, spinal muscular atrophy; HC, healthy control; *N*, number; SD, standard deviation; BMI, body mass index; *SMN2, survival of motor neuron 2* gene; NIV, non-invasive ventilation; PEG, percutaneous endoscopic gastrostomy; HFMSE, Hammersmith Functional Motor Scale Expanded; RULM, Revised Upper Limb Module; BDI, Beck Depression Inventory; FSS, Fatigue Severity Scale; SF-36, 36-Item Short Form Health Survey

### Overall burden of autonomic symptoms in SMA patients vs. healthy controls

**Fig. 1:**
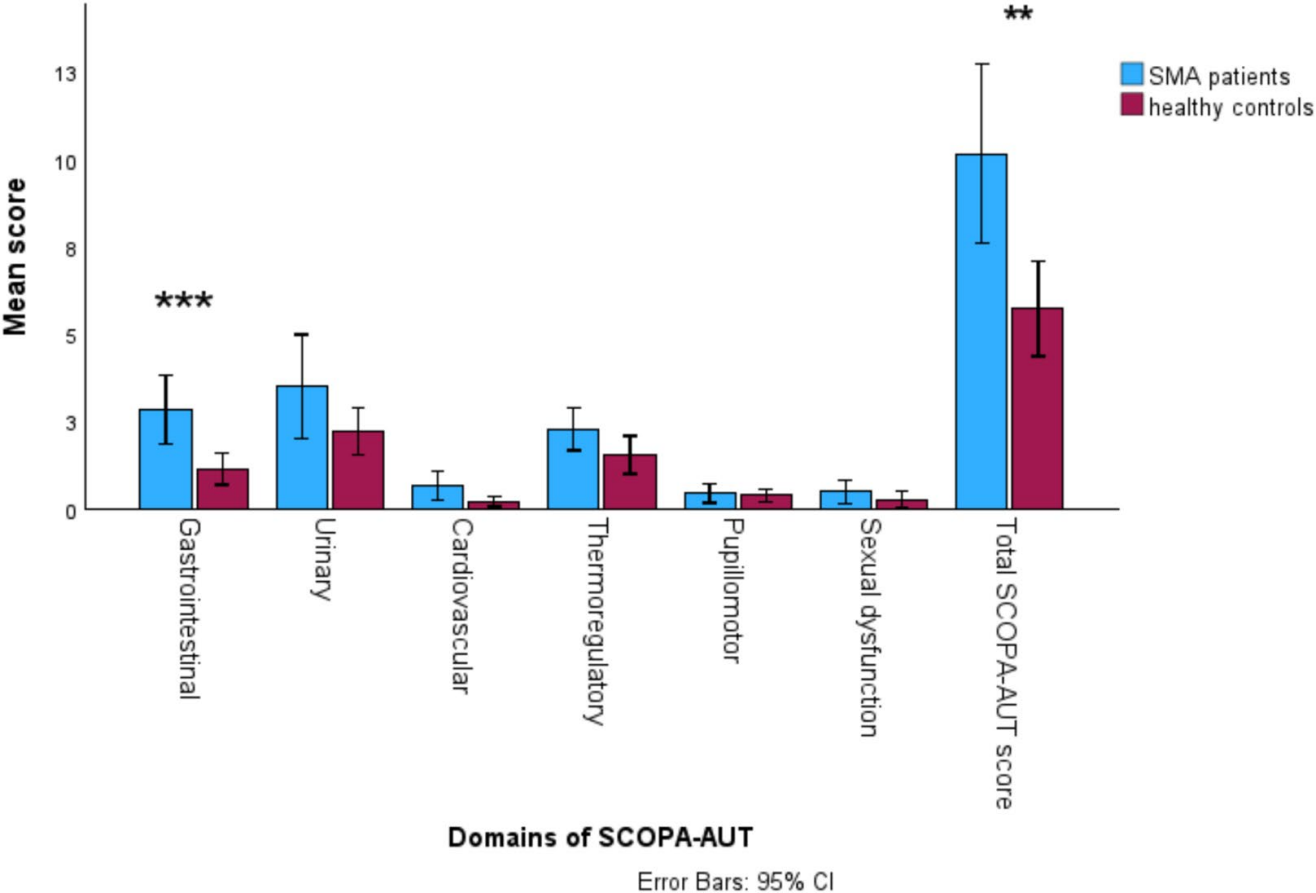
Autonomic function and its domains in adult SMA patients vs. healthy controls SMA, spinal muscular atrophy, SCOPA-AUT, SCales for Outcomes in Parkinson’s disease-Autonomic Dysfunction; CI, confidence interval; ***, *p* < 0.001 >; **, *p* < 0.01

The differences in the domains of the autonomic function between SMA patients and HC are shown in Fig. 1. SMA patients scored significantly worse in the gastrointestinal domain (2.9 ± 2.9 vs 1.1 ± 1.5, *p* < 0.001) and in the total SCOPA-AUT score (9.8 ± 7.3 vs. 5.7 ± 4.4, *p* = 0.002), compared to HC. No differences between SMA patients and HC were found in following domains: urinary, cardiovascular, thermoregulatory, pupillomotor, and sexual.

### Association of sociodemographic/clinical characteristics and autonomic dysfunction in SMA patients

Female SMA patients had significantly worse scores in the cardiovascular (1.0 ± 1.6 vs. 0.2 ± 0.5, *p* = 0.023) and sexual domain (0.9 ± 1.3 vs. 0.2 ± 0.6, *p* = 0.041) compared to male patients (Supplemental Fig. 1A). SMA patients receiving risdiplam exhibited worse scores in the gastrointestinal (4.4 ± 2.9 vs. 1.8 ± 2.5, *p* = 0.007), urinary (5.5 ± 6.6 vs. 2.4 ± 2.6, *p* = 0.042), and cardiovascular domain (1.1 ± 1.9 vs. 0.3 ± 0.5, *p* = 0.046), as well as higher total SCOPA-AUT score (14.8 ± 9.7 vs. 7.2 ± 4.4, *p* = 0.002) compared to those receiving nusinersen (Supplemental Fig. 1B). SMA patients with scoliosis had significantly worse scores in the gastrointestinal domain (3.5 ± 2.9 vs. 0.9 ± 1.9, *p* = 0.011) compared to those without scoliosis (Supplemental Fig. 1C). SMA patients with fewer than four *SMN2* gene copies had significantly worse scores in the thermoregulatory domain (2.8 ± 1.9 vs. 1.6 ± 1.3, *p* = 0.030) compared to those with four or more copies (Supplemental Fig. 1D). Patients with SMA type 2 had significantly worse scores in the gastrointestinal (3.8 ± 2.7 vs. 1.9 ± 2.4, *p* = 0.018) domain, compared to SMA type 3 patients (Supplemental Fig. 1E). In addition, SMA patients with depression had significantly worse scores in the cardiovascular domain (1.4 ± 1.8 vs. 0.3 ± 0.8, *p* = 0.014) compared to those without depression (Supplemental Fig. 1F). We observed no differences in subgroup analysis regarding ambulatory status, spondylodesis, and presence of fatigue. A weak negative correlation between the total SCOPA-AUT score and HFMSE was observed, which approached statistical significance (rho = − 0.30, *p* = 0.054). No correlation was observed between the total SCOPA-AUT score and RULM.

### Association of quality of life with autonomic dysfunction in SMA patients

**Fig. 2:**
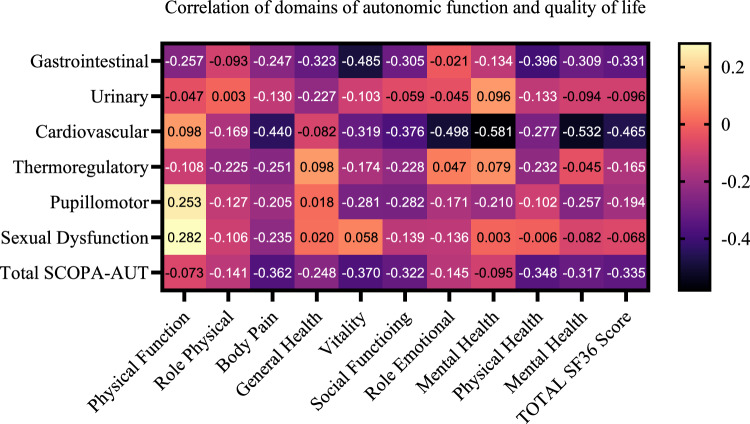
Heatmap of correlation of autonomic function domains and quality of life in SMA patients. SCOPA-AUT, Scale for Outcomes in Parkinson’s Disease-Autonomic Dysfunction; SF-36, Short Form-36

Figure 2 shows the correlation of autonomic function domains and QoL in SMA patients. The total SCOPA-AUT score showed a significant negative correlation with the following QoL domains: body pain (rho = − 0.36, *p* = 0.017), vitality (rho = − 0.37, *p* = 0.015), physical composite score (rho = − 0.348, *p* = 0.022), mental composite score (rho = − 0.32, *p* = 0.039), and finally, total SF-36 score (rho = − 0.33, *p* = 0.028).

### ECG findings in adults with SMA

**Fig. 3:**
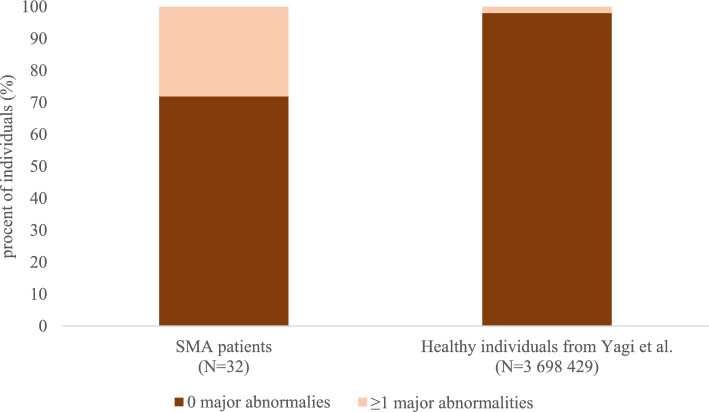
Frequencies of major abnormalities in our cohort of SMA patients in comparison to the healthy individuals from the recent publication by Yagi et al. [16]. SMA, spinal muscular atrophy; *N*, number

**Fig. 4:**
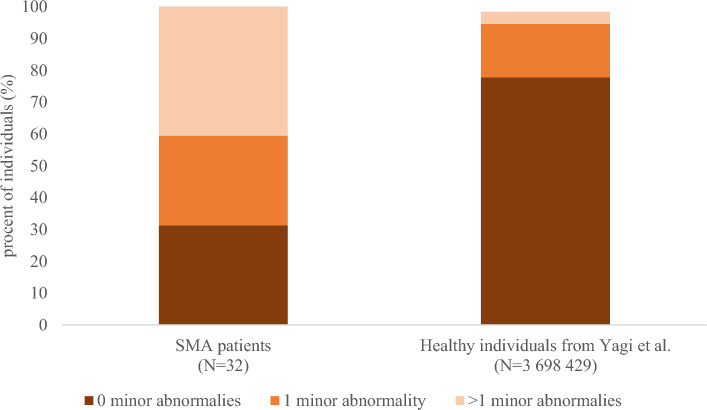
Frequencies of minor abnormalities in our cohort of SMA patients in comparison to the healthy individuals from the recent publication by Yagi et al. [16]. SMA, spinal muscular atrophy; *N*, number

Thirty-two ECGs were available for further analysis. Figures 3 and 4 present the frequencies of major and minor abnormalities in our SMA cohort in comparison to healthy individuals from the recent publication by Yagi et al. [16]. Table 2 shows the frequencies of rhythm, axis, atrial, atrioventricular, QRS conduction, QRS complex, repolarization and hypertrophy ECG abnormalities in our SMA patients. The most common ECG abnormalities involved the QRS complex, followed by repolarization and rhythm abnormalities (Table 2).

**Table 2:** Frequencies of ECG abnormalities in our adult SMA cohort

ECG abnormalities	SMA patients (*N* = 32)
Rhythm (*N*, %)	
Sinus rhythm	22, 68.7
Sinus arrhythmia	4, 12.5
Sinus tachycardia (86–100 bpm)	2, 6.3
Sinus tachycardia (> 101 bpm)	4, 12.5
Axis (*N*, %)	
Normal	25, 78.1
Left deviation	6, 18.8
Undetermined axis deviation	1, 3.1
Atrial (*N*, %)	
Normal	30, 93.7
High P	2, 6.3
Atrioventricular (*N*, %)	
Normal	29, 90.6
Pre-excitation/WPW	1, 3.1
Short PQ	2, 6.3
QRS conduction (*N*, %)	
Normal	24, 75.0
High R amplitude	2, 6.3
Delayed R progression	2, 6.3
Borderline Q	3, 9.3
Abnormal Q	1, 3.1
QRS complex (*N*, %)	
Normal	18, 56.3
Incomplete RBBB RsR’	7, 21.9
Left anterior hemiblock	3, 9.3
Unspecific intraventricular block	4, 12.5
Repolarization (*N*, %)	
None	21, 65.6
Abnormal T wave	1, 3.1
Early depolarization	3, 9.3
Non-specific ST-T change	5, 15.6
QTc > 440 ms for men/460 ms for women	1, 3.1
T inversion (> 0.5 mV)	1, 3.1
Hypertrophy (*N*, %)	
None	30, 93.8
High amplitude R wave: right	1, 3.1
Left with ST/T changes	1, 3.1

ECG, electrocardiogram; *N*, number; bpm, beats per minute; WPW, Wolff–Parkinson–White syndrome; Incomplete RBBB RsR’, incomplete right bundle branch block with RsR’; ms, milliseconds; mV, millivolt

Nearly one-third of SMA patients exhibited at least one major ECG abnormality, while more than two-thirds had at least one minor abnormality. The presence of at least one major abnormality was more frequent in patients with SMA type 2 (rho = − 0.39, *p* = 0.027) and in those who were non-ambulatory (rho = − 0.39, *p* = 0.027). Furthermore, a moderate negative correlation between the presence of at least one major abnormality and age at the time of testing was observed (rho = − 0.45, *p* = 0.009). In contrast, minor abnormalities were not associated with any of the sociodemographic or clinical characteristics (*p* > 0.05). In addition, we observed a weak negative correlation between the HFMSE score and both the number of major (rho = − 0.36, *p* = 0.043) and minor abnormalities (rho = − 0.35, *p* = 0.05). No correlation was found between the cardiovascular domain of the SCOPA-AUT or total SCOPA-AUT score and the presence of either major or minor abnormalities (*p* > 0.05).

## Discussion

To the best of our knowledge, this is the first study to assess the burden of autonomic symptoms in adults with SMA and to compare these findings with age- and sex-matched HC. Our adult SMA cohort exhibited a significantly higher overall burden of autonomic symptoms compared to HC, with the most pronounced differences observed in the gastrointestinal domain. More severely affected SMA patients (SMA type 2 patients, with scoliosis, fewer *SMN2* gene copies, and lower HFMSE scores) reported a greater burden of autonomic symptoms compared to those with milder phenotypes.

Previous studies investigating non-motor symptoms and autonomic dysfunction in SMA patients are rare and have primarily focused on SMA type 1, as these patients exhibit the most severe SMN deficiency. Hachiya et al. showed an increased heart rate variability and suboptimal autonomic regulation of vascular responses to temperature in two invasively ventilated infants with SMA type 1 [20]. Araujo et al. reported two cases of infants with SMA type 1 in whom a distal necrosis of fingers and toes due to autonomic dysfunction developed [9]. Arai et al. evaluated finger cold-induced vasodilatation, sympathetic skin response, and R-R interval variation in ten patients with SMA (seven with SMA type 1, two with type 2, and one with type 3) and concluded that some SMA patients have autonomic dysfunction, especially sympathetic nerve hyperactivity [8].

In our cohort, SMA patients showed the greatest impairment in the gastrointestinal domain of autonomic function. In preclinical studies, Gombash et al. demonstrated that SMN deficiency led to constipation, delayed gastric emptying, slowed intestinal transit, and reduced colonic motility in mice, despite normal activity levels and unaltered food and water intake. Notably, these functional disturbances occurred in the absence of gross anatomical or histopathological abnormalities, suggesting that SMN depletion may disrupt intrinsic neural regulation of gastrointestinal motility mediated by the enteric nervous system [21]. Another possible explanation for the worse scores in the gastrointestinal domain in our SMA patients in comparison to the HC group is that our SMA cohort included patients treated with risdiplam. Gastrointestinal symptoms (such as bloating, discomfort, abdominal pain and nausea) are among the most commonly reported side effects of risdiplam [22]. However, none of the patients in our study reported experiencing any relevant side effects at the time of assessment. Contrary to our findings, a previous study involving 70 adults with SMA reported a low burden of non-motor symptoms relative to HC. However, the questionnaire used in that study did not specifically assess autonomic symptoms [23]. In amyotrophic lateral sclerosis (ALS), another motor neuron disease, autonomic dysfunction has also been reported, most commonly affecting the gastrointestinal and urinary system [24]. Overall, our findings support the hypothesis that even adults with milder SMA phenotypes exhibit substantial autonomic dysfunction.

ECG abnormalities were common in our SMA cohort, affecting nearly one-third with major and over two-thirds with minor findings. Compared to the largest population-based study on healthy Japanese individuals to date, conducted by Yagi et al. and including over 3.6 million participants [16], these frequencies are markedly higher in our SMA cohort. In the study of Yagi et al., only 2% of individuals presented with major ECG abnormalities and 20% with minor ones. The most frequently observed abnormalities in our SMA cohort involved the abnormal QRS complex, repolarization, rhythm and QRS conduction. Previous preclinical studies showed microscopic abnormalities of the cardiac autonomic nervous system, including reduced neuronal branching and presence of thinner cardiac sympathetic nerves in murine SMA models [25, 26]. In clinical studies, cardiovascular involvement has most commonly been described as non-motor manifestations in SMA [27]. In SMA type 1, both structural cardiac defects (such as atrial and ventricular septal defects) and conduction abnormalities (such as prolonged atrioventricular conduction times and bradycardia) have been observed [27, 28]. A study by Djordjevic et al. investigating 42 children and adolescents with SMA type 2 and 3 found a low prevalence of cardiac abnormalities, reporting only one case of mitral valve prolapse, seven cases of sinus tachycardia, and one case of prolonged P-R interval [29]. The discrepancy between their findings and ours may be explained by the significantly younger age of participants in their study, with the oldest patient being about 14 years old. In contrast, our study focused on adult SMA patients, in whom cumulative disease burden may contribute to a higher frequency of ECG abnormalities. In line with this, we observed a moderate negative correlation between disease severity and the presence of ECG abnormalities. Interestingly, we also identified a moderate negative correlation between age at the time of testing and the presence of at least one major abnormality. This finding is in contrast to trends observed in the general population, where the risk of cardiac pathology and ECG abnormalities typically increases with age [30]. As disease-modifying therapies allow more patients with severe forms of SMA to reach adulthood, autonomic dysfunction, a non-motor feature of SMA, may become an increasingly important focus for both future research and clinical care.

The burden of autonomic symptoms had negative impact on QoL in our SMA cohort. A possible explanation could be that these symptoms (such as gastrointestinal complaints) may lead to physical discomfort and contribute to social isolation. Similarly, circulatory and thermoregulatory disturbances might result in anxiety and a perceived loss of control over vegetative symptoms (e.g., palpitation, sweating). In line with this, our SMA patients with depression had significantly worse scores in the cardiovascular domain of the SCOPA-AUT compared to those without depression. To the best of our knowledge, the relationship between the burden of autonomic symptoms and QoL has not been systematically investigated in SMA before. These findings underscore the importance of routinely assessing autonomic symptoms in clinical practice. Raising awareness and offering symptomatic treatment may help improve QoL of adults with SMA.

One of the limitations of our study arises from its monocentric design and relatively small cohort size. In addition, the burden of autonomic symptoms was assessed via self-report, which may introduce re-call and reporting bias. The SCOPA-AUT questionnaire was originally developed to evaluate autonomic symptoms in patients with Parkinson’s disease and multiple system atrophy, and has not been validated for use in SMA. We did not include any functional autonomic testing in our study, which should be addressed in future studies. Furthermore, the comparison of autonomic function between patients with severe motor impairment and HC makes it challenging to distinguish which autonomic features are directly driven by SMN deficiency outside motor neurons and which are secondary to reduced mobility, deconditioning, or other consequences of severe muscle weakness. Future studies could address this limitation by including a control group with a comparable degree of physical disability due to a different disease mechanism, such as chronic muscle disease, stroke, or ALS. Prospective, multicenter studies are needed to better understand the prevalence of autonomic symptoms and the factors associated with them in adults with SMA.

In conclusion, adults with SMA demonstrate a substantial burden of autonomic symptoms, especially those with more severe disease. This burden significantly compromises patients’ QoL. Further studies are needed to better understand autonomic dysfunction in this population.

## Supplementary Information

Below is the link to the electronic supplementary material.

Supplementary file1 (DOCX 3622 KB)

## Data Availability

De-identified data will be shared on reasonable request with any qualified investigator.
