# Supplementary material for: Beyond motor neurons: autonomic dysfunction and ECG findings in adults with 5q-spinal muscular atrophy
Source: J Neurol. 2025 Oct 14;272(10):694. doi: 10.1007/s00415-025-13446-w (PMC12521311; doi:10.1007/s00415-025-13446-w)
Supplement: Supplementary file 1 — Supplementary file1 (DOCX 3622 KB) [file 415_2025_13446_MOESM1_ESM.docx]

Supplemental table 1. Modified version of the classification system of ECG abnormalities, adapted from Yagi et al. [16]

| Categorization | Diagnosis |
| --- | --- |
| Minor ECG abnormality | A-V block (Wenckebach´s phenomenon)  Abnormal T wave  Borderline Q wave  Complete RBBB  Early depolarization  High amplitude R wave: right  High left ventricular voltage (without ST-T change)  High P wave amplitude  High T wave amplitude  Incomplete RBBB  Indetermined axis deviation  Intermittent aberrant A-V conduction  Intermittent RBBB  Left anterior hemiblock  Left/extreme QRS axis deviation  Low QRS amplitude  P-R interval ≥ 0.22 sec  Persistent supraventricular rhythm  Premature atrial contraction  Premature ventricular contraction  R-R´ Pattern  Right axis deviation  Short PQ interval  Sinus arrhythmia  Sinus bradycardia (40-44 bpm)  Sinus tachycardia (86-100 bpm)  ST elevation  Non-specific ST-T change  Wandering atrial pacemaker |
| Major ECG abnormality | A-V dissociation  Abnormal Q wave  Atrial fibrillation  Atrial flutter  Brugada type ST elevation  Combination of left anterior hemiblock and complete RBBB  Complete A-V block  High amplitude R waves (biventricular)  High left ventricular voltage (with ST-T change)  Intraventricular block  Left bundle branch block  Mobitz Type II A-V block  Multifocal premature atrial contraction  Multifocal premature ventricular contraction  Negative T wave (≥0.5mV)  Partial A-V block (2:1)  Poor R progression  S-A block  Sinus bradycardia (≤39bpm)  Sinus tachycardia (≥101bpm)  ST-T depression (horizontal/downsloping)  Supraventricular tachycardia  Ventricular tachycardia  Wolf-Parkinson-White syndrome |

ECG, electrocardiogram; A-V, atrioventricular; S-A, sinoatrial; RBBB, right bundle branch block

Supplemental figures 1. Association of sociodemographic/clinical characteristics and autonomic dysfunction in SMA patients – subgroup analysis

A Subgroup analysis stratified by sex


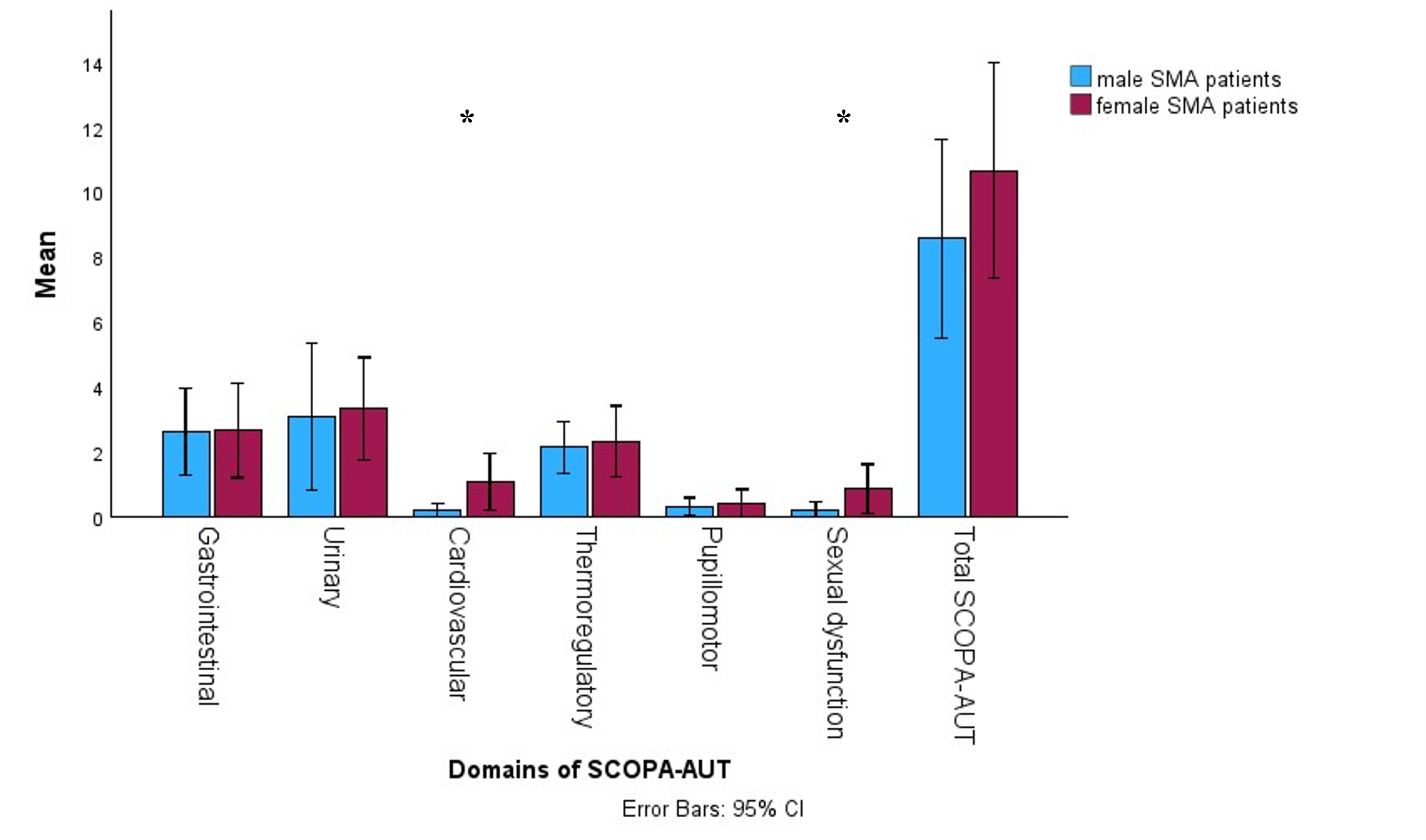


B Subgroup analysis stratified by therapy regime at the moment of testing


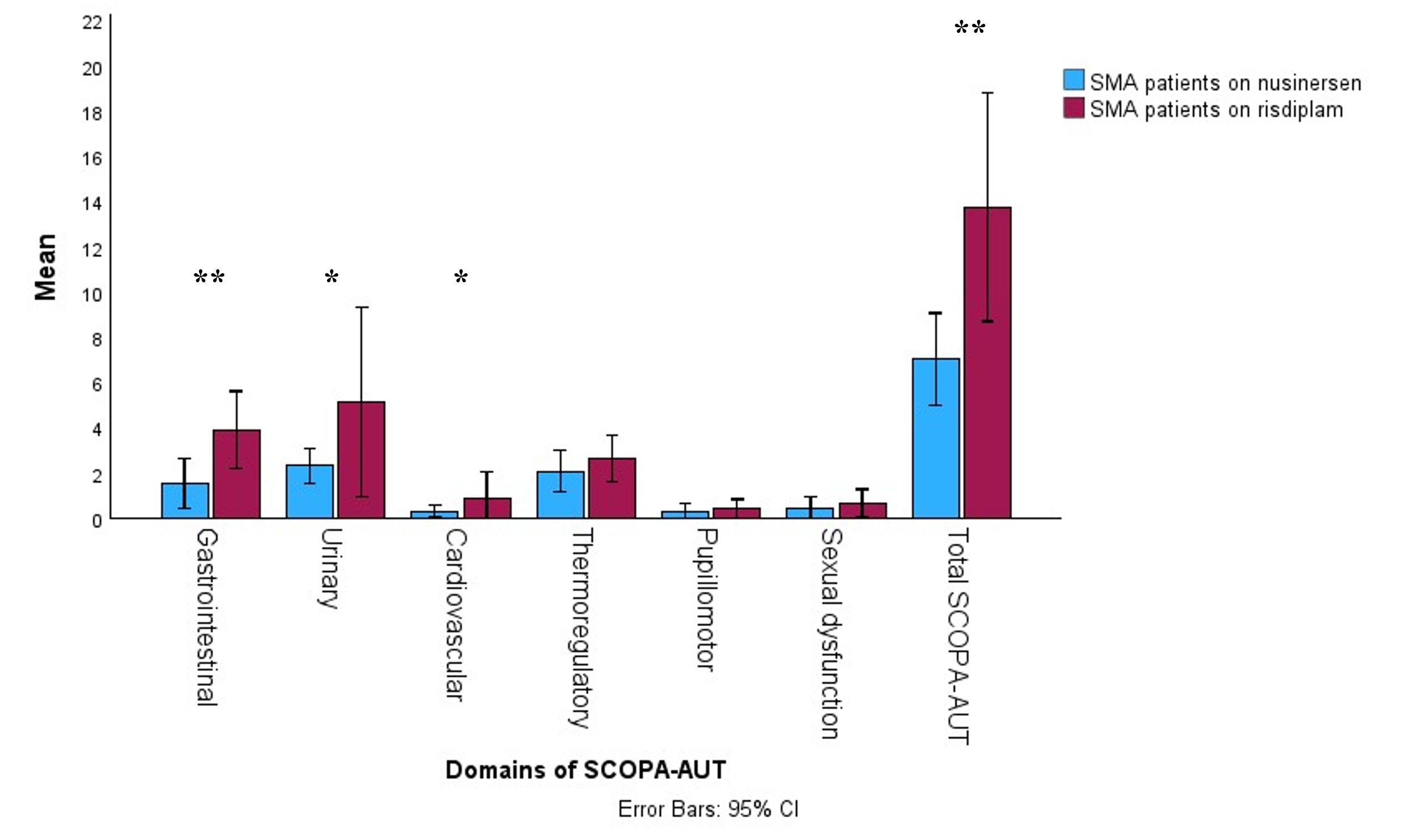


C Subgroup analysis stratified by presence of scoliosis


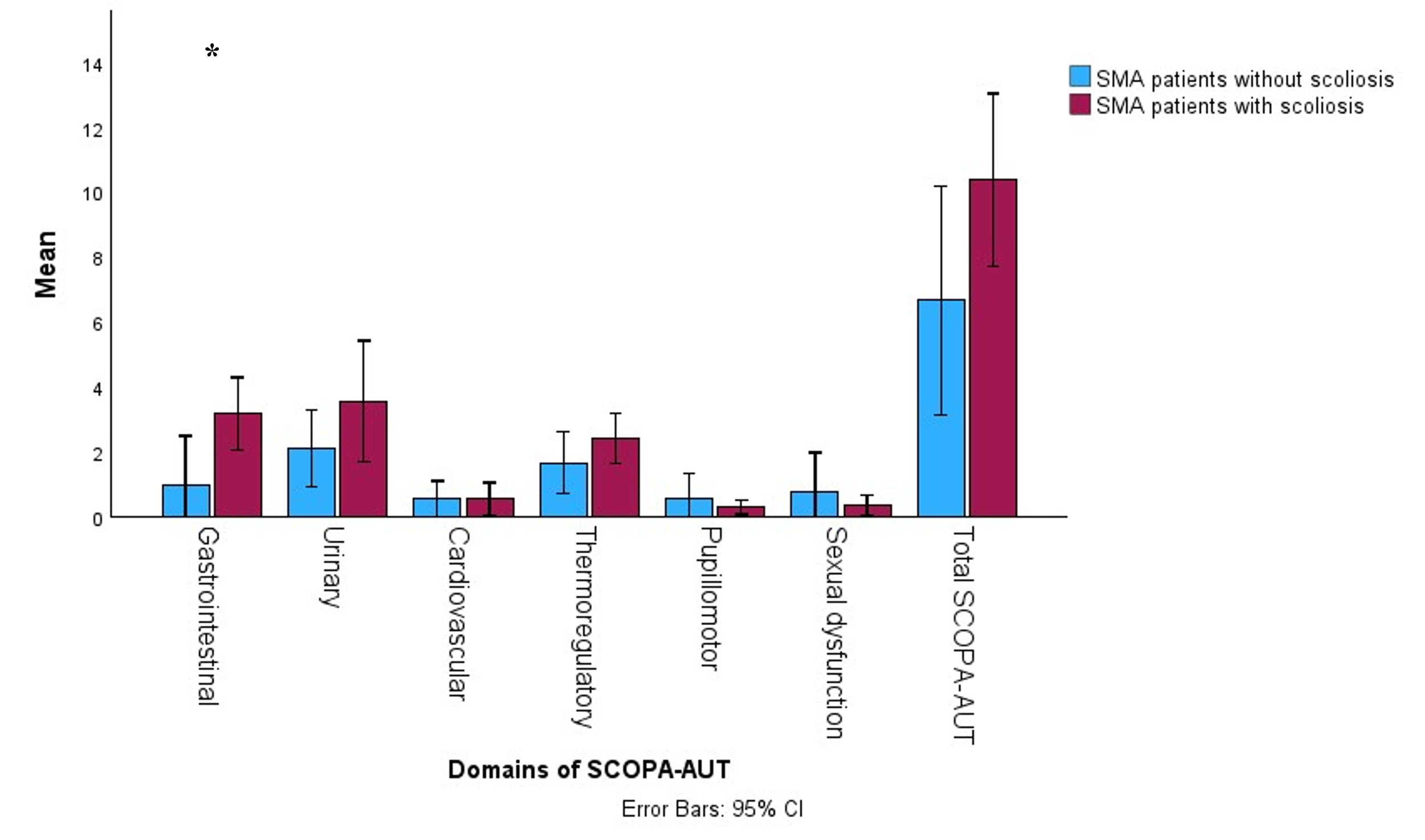


D Subgroup analysis stratified by *SMN2* copy number


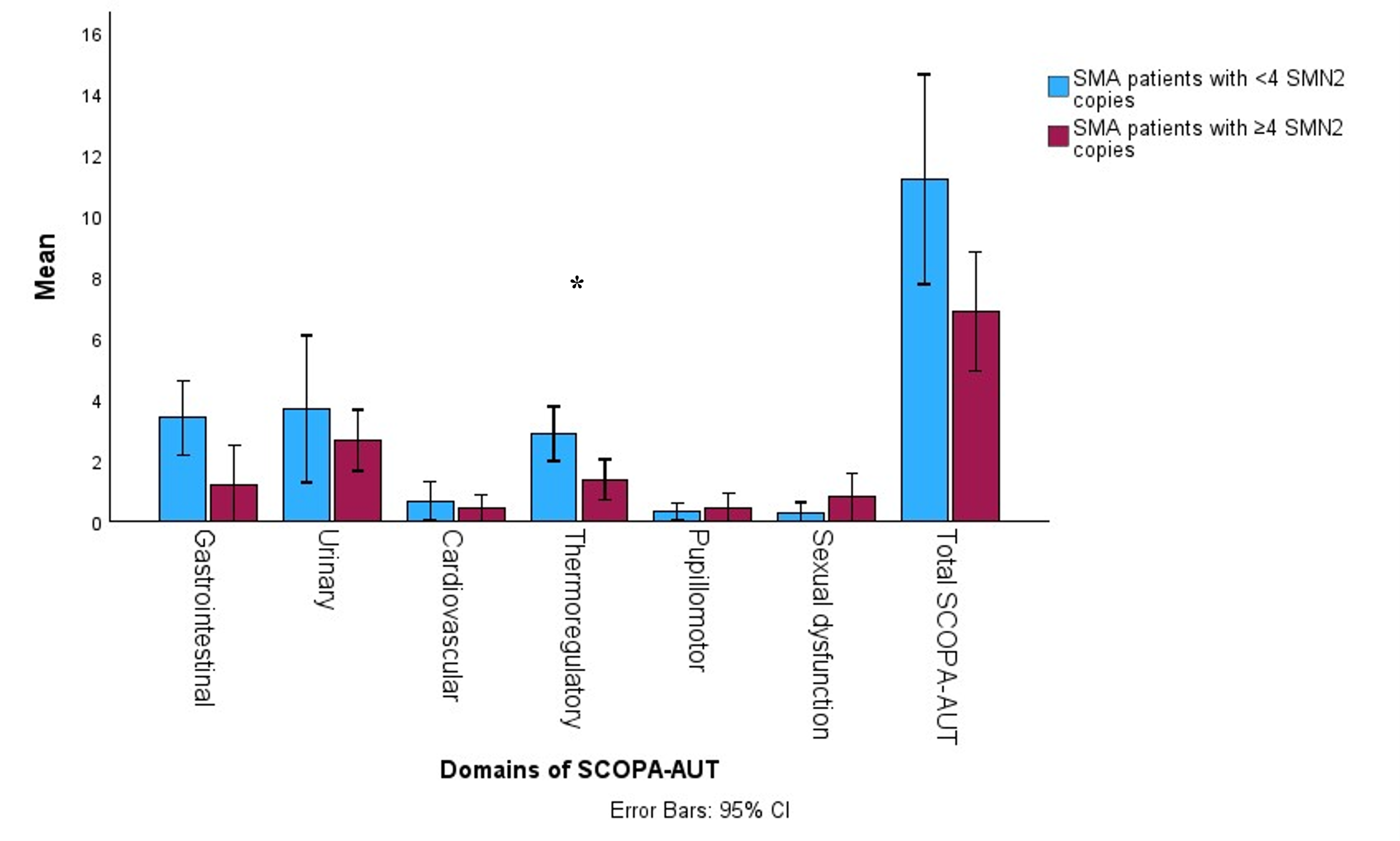


E Subgroup analysis stratified by SMA type


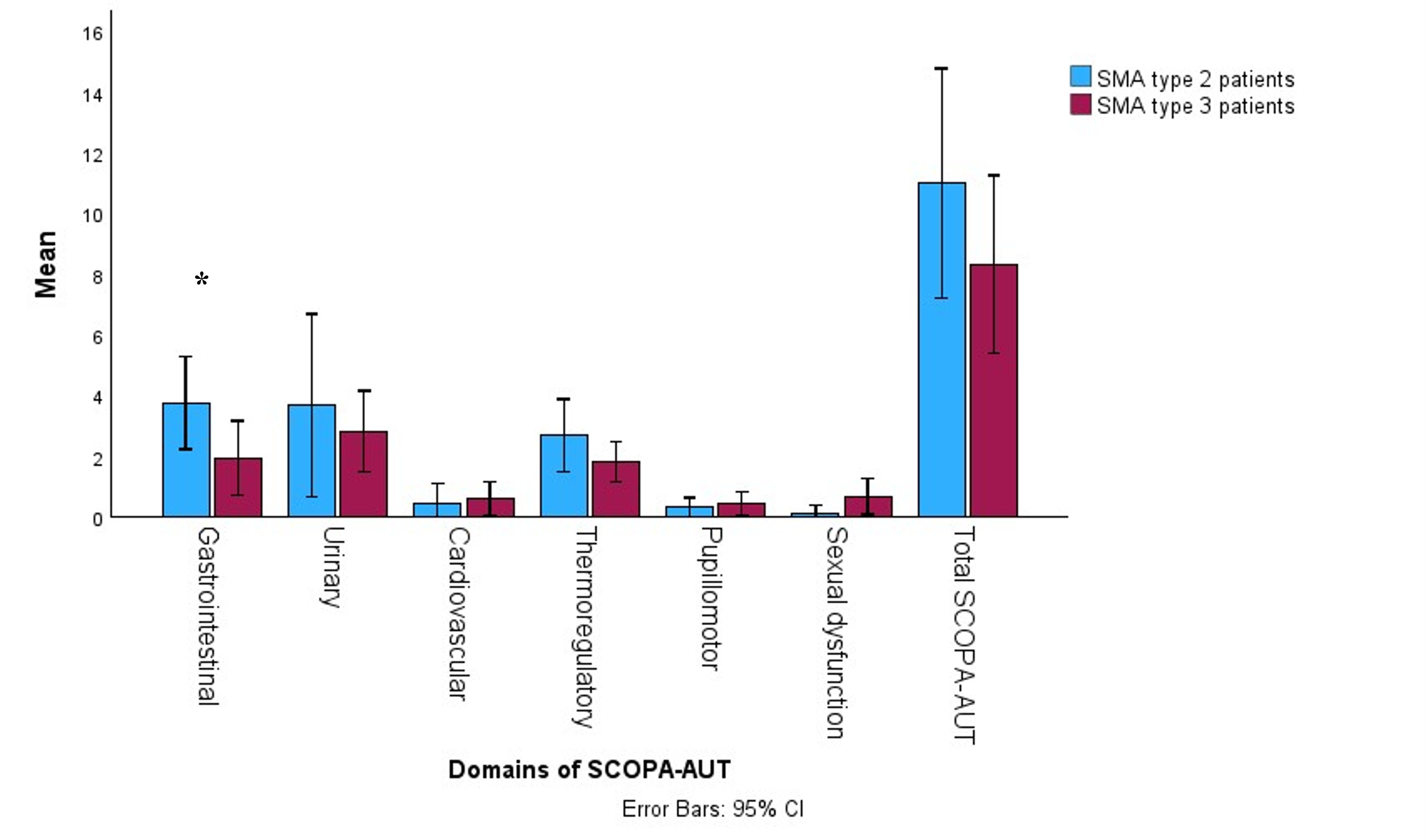


F Subgroup analysis stratified by presence of depression


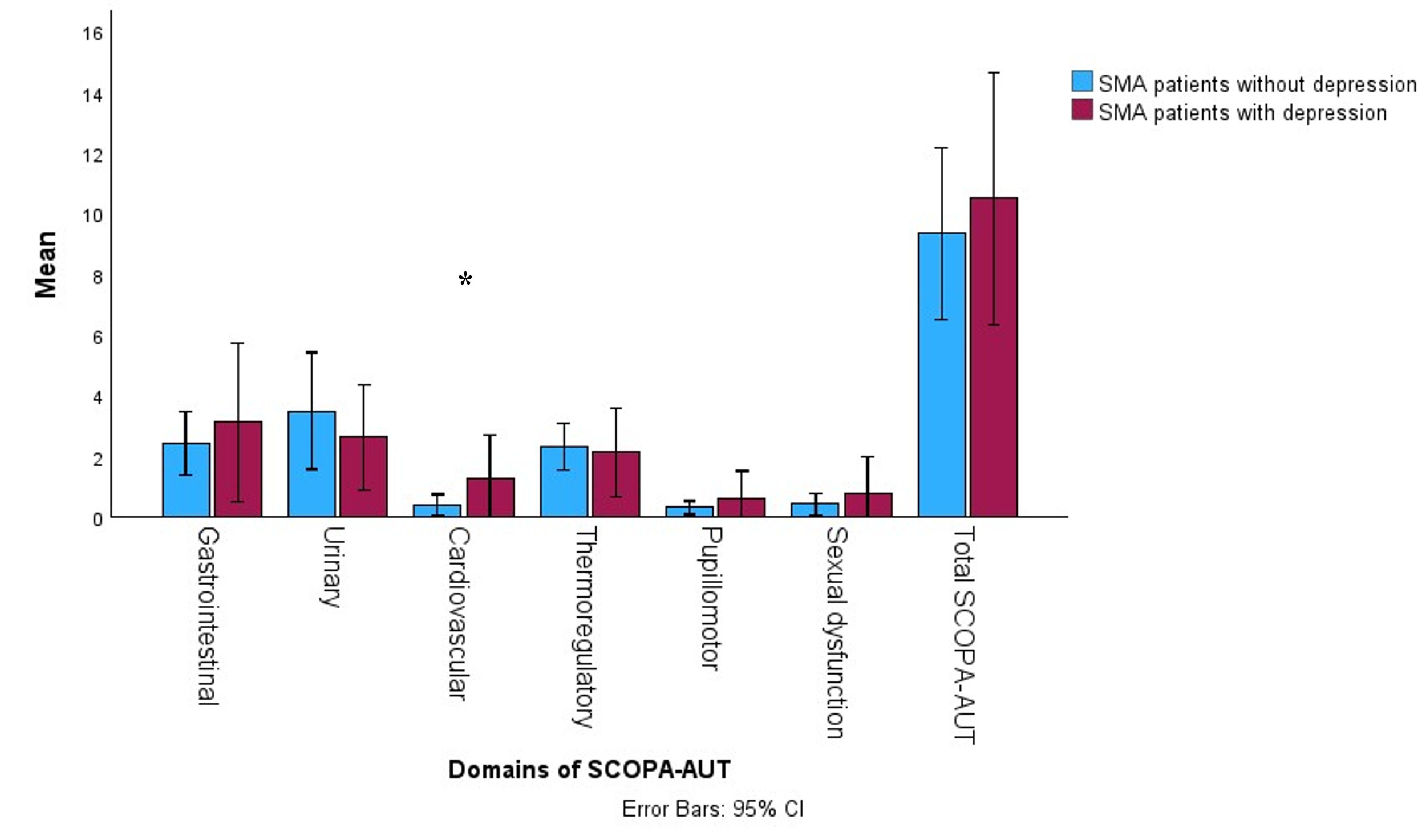


SMA, spinal muscular atrophy; SCOPA-AUT, SCales for Outcomes in Parkinson’s disease – Autonomic Dysfunction; CI, Confidence Interval; *SMN2*, *survival of motor neuron 2* gene **, p<0.01>; *, p<0.05
